# Validation of Clinical Observations of Mastication in Persons with ALS

**DOI:** 10.1007/s00455-015-9685-3

**Published:** 2016-01-23

**Authors:** Meg Simione, Erin M. Wilson, Yana Yunusova, Jordan R. Green

**Affiliations:** Speech and Feeding Disorders Lab, MGH Institute of Health Professions, 36 1st Avenue, Boston, MA 02129 USA; Waisman Center, University of Wisconsin, Madison, WI USA; Department of Speech Language Pathology, University of Toronto, Toronto, ON Canada

**Keywords:** Dysphagia, Clinical assessment, Mastication, Kinematics, Amyotrophic lateral sclerosis, Deglutition, Deglutition disorders

## Abstract

Amyotrophic lateral sclerosis (ALS) is a progressive neurological disease that can result in difficulties with mastication leading to malnutrition, choking or aspiration, and reduced quality of life. When evaluating mastication, clinicians primarily observe spatial and temporal aspects of jaw motion. The reliability and validity of clinical observations for detecting jaw movement abnormalities is unknown. The purpose of this study is to determine the reliability and validity of clinician-based ratings of chewing performance in neuro-typical controls and persons with varying degrees of chewing impairments due to ALS. Adults chewed a solid food consistency while full-face video were recorded along with jaw kinematic data using a 3D optical motion capture system. Five experienced speech-language pathologists watched the videos and rated the spatial and temporal aspects of chewing performance. The jaw kinematic data served as the gold-standard for validating the clinicians’ ratings. Results showed that the clinician-based rating of temporal aspects of chewing performance had strong inter-rater reliability and correlated well with comparable kinematic measures. In contrast, the reliability of rating the spatial and spatiotemporal aspects of chewing (i.e., range of motion of the jaw, consistency of the chewing pattern) was mixed. Specifically, ratings of range of motion were at best only moderately reliable. Ratings of chewing movement consistency were reliable but only weakly correlated with comparable measures of jaw kinematics. These findings suggest that clinician ratings of temporal aspects of chewing are appropriate for clinical use, whereas ratings of the spatial and spatiotemporal aspects of chewing may not be reliable or valid.

## Introduction

Difficulties with mastication and swallowing occur frequently in persons with amyotrophic lateral sclerosis (ALS). ALS eventually weakens the muscles of mastication and swallowing even in persons who primarily present with only spinal muscle weakness early in the disease [1–3]. Impaired mastication can significantly increase the risk for choking, aspiration, and malnutrition [4, 5], which can result in hospitalizations, placement of a gastronomy tube, and decreased quality of life [6]. Clinicians, therefore, routinely evaluate mastication to determine swallowing safety, to maximize nutrition, and to monitor disease progression. When evaluating mastication, clinicians observe the spatial (e.g., jaw excursions, jaw movement patterns) and temporal (e.g., duration of the chewing sequence, rate of chewing) aspects of mandibular movements and their efficiency for breaking down food [7, 8]. Clinical assessment is predicated on the assumption that deviations in spatial and temporal movement patterns of the mandible decrease the safety and efficiency of mastication.

Despite its importance for safety, health, and quality of life, the options for assessing chewing motor skills are currently very limited. One of the few instrumentation approaches available in hospital settings, videofluoroscopic swallowing studies, is not appropriate for some patients because of risks associated with radiation exposure. Moreover, the existing clinical scales have primarily been designed to evaluate chewing skills in children [9–11], or to examine feeding or swallowing rather than chewing. For example, the standard clinical oral motor examination often includes the evaluation of jaw range of motion, speed, and strength, but not specifically while chewing. Other scales solely assess functional aspects of eating [12, 13]. Only one item of the ALS Functional Rating Scale—revised [14], for example, targets oral intake skills and it is narrowly focused on determining feeding status, ranging from full oral feedings to receiving nutrition via alternative methods. In the absence of validated protocols and scales, clinicians rely almost exclusively on visual observation of chewing. It is unknown, however, if such observations are sensitive enough to detect changes to temporal and spatial aspects of mandibular movements with disease progression.

In this study, we investigate the reliability of clinicians’ ratings of chewing performance in persons with ALS and neuro-typical controls and the validity of those measures using biomechanic-based measures of chewing performance. High-speed digital cameras recorded the movements of chin markers in three dimensions, which were used to obtain accurate and detailed information about jaw movement displacement, speed, and performance variability during chewing [15, 16]. This technology has been used to detect gains in mandibular control in early development [17–19] and declines in mandibular control with neurodegenerative disease. For example, a recent study on speech motor decline in persons with ALS observed declines in jaw movement speed prior to changes in speaking rate and speech intelligibility [20, 21]. Because of its ability to detect small, subtle movements that are not easily discerned through observations, motion capture is likely to be more sensitive to change than observation-based judgments. The goals of this study are to determine (1) the reliability of clinician ratings of chewing performance in persons with ALS and neuro-typical controls, and (2) the validity of those measures using biomechanic-based measures of chewing performance using three-dimensional (3D) optical motion capture.

## Methods

### Participants

Participants were 19 individuals with ALS and 10 neuro-typical controls. Neuro-typical controls were included to ensure a representation of normal to severely disordered chewing. The mean age of participants with ALS was 58.26 (12.19) years with a range of 40–77 years; 8 of the individuals were female and 11 were male. The site of onset varied—5 people had bulbar onset, 13 had spinal onset, and 1 was unknown. The participants had a wide range of severity of bulbar symptoms with a mean speaking rate on the sentence intelligibility test (SIT) [22] of 159.02 (53.84) words per minute (wpm) with a range of 29.09-262.95 wpm. The average speaking rate for the SIT sentences for healthy talkers was reported to be 180 wpm [23]. The mean intelligibility score on the SIT was 92.54 (12.56) % with a range of 56.75–100 % intelligibility.

### Task

All participants were seated in a comfortable chair with head support and offered a solid consistency food of 3–5 Cheerios (General Mills). While chewing the solid food, full-face videos were recorded while simultaneously collecting 3D motion capture information from the jaw. The full-face video recordings were used for observation-based judgments by five experiences speech-language pathologists (SLPs) and the motion capture information was used for the kinematic analysis.

### Clinician Ratings of Chewing Performance

The five speech-language pathologists served as the raters. The SLPs all worked in an acute care hospital, and evaluated and treated patients with dysphagia as part of their daily caseload. The mean years of experience working was 10.6 (8.73) years with a range of 2–25 years. Each of the SLPs viewed 35 randomized videos using online presentation software, Limesurvey [24]. The resolution of the video was 720 × 480 pixels. The SLPs were provided with rating instructions and were allowed to re-watch each video as many times as needed to answer the following four items: (1) How many seconds is the chewing sequence?, (2) How many chewing cycles are in the chewing sequence?, (3) Rate the person’s range of motion of the jaw using a scale ranging from 1 to 5 (See Table 1), (4) How consistent is the chewing pattern using a scale ranging from 1 to 4 (See Table 1)? The SLPs were provided with additional information about each of the items including instructions about how to determine the beginning and ending of the chewing sequence. Table 1 shows the observation-based judgments used in the online survey and the corresponding names of the kinematic variables.

**Table 1:** Survey questions and kinematic correlate

Survey question	Kinematic correlate
How many seconds is the chewing sequence?	Duration of the chewing sequence (s)
How many chewing cycles are in the chewing sequence?	Number of cycles in the chewing sequence
Rate the person’s range of motion of the jaw (Rating scale from 1–5 below)	Working space of mandibular movements (mm^3^)
Severely reduced	
Reduced	
Within normal limits	
Exaggerated	
Severely exaggerated	
How consistent is the chewing pattern? (Rating scale from 1–4 below)	Cycle-to-cycle spatiotemporal variability
Within normal limits	
Mildly inconsistent	
Moderately inconsistent	
Severely inconsistent	

### Obtaining Kinematics Using a 3-Dimensional Motion Capture System

**Fig. 1:**
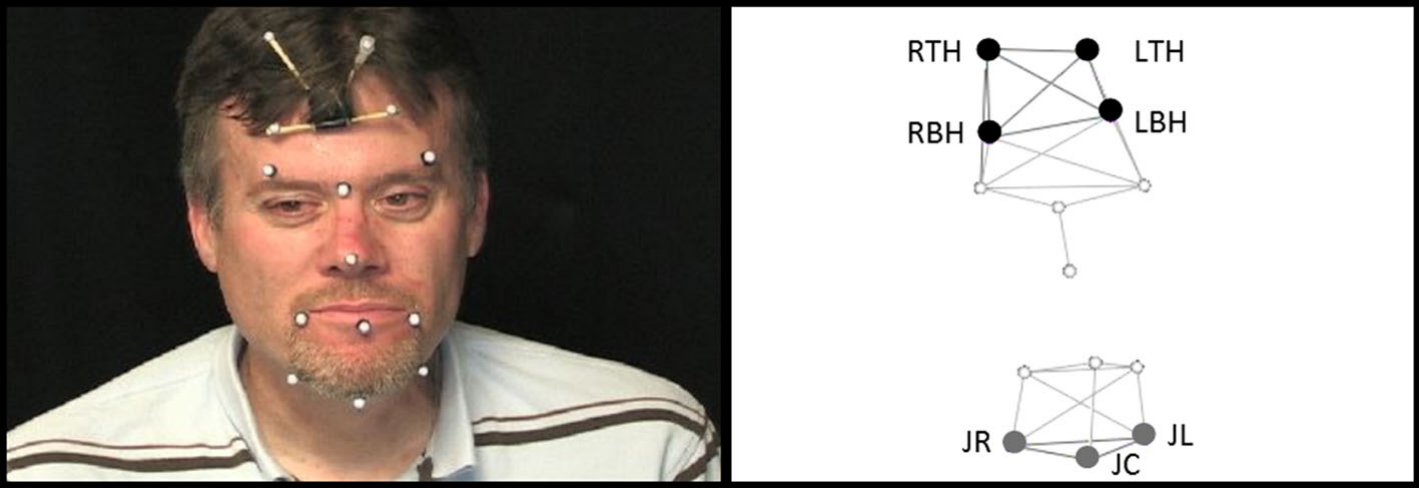
The marker placement is shown on the *left*. On the *right* is the corresponding marker set of the jaw (shown in *gray*) and head (shown in *black*) in 3-dimensional space. The markers that are not labeled were not used for the analyses in this study. *JR* jaw right; *JL* jaw left; *JC* jaw central; *RTH* right top head; *RBH* right bottom head; *LTH* left top head; *LBH* left bottom head

Jaw movements during chewing were registered at 120 frames per second using 3D optical motion capture [25] with eight cameras. The movement data were digitally low-pass filtered (*f*_lp_ = 10 Hz) using a zero-phase shift forward and reverse digital filter (Butterworth, 8 pole). One reflective spherical marker was placed on the center of the jaw gnathion (JC) and two markers were placed to the right (JR) and left (JL). For the analysis, only the JR was used because prior work suggests that flesh-point markers located to either the left or right of JC are less prone to error due to less movement of flesh [16]. A 4-marker array was placed on the forehead to remove the translation and rotation components of head movement from the jaw movements resulting in jaw movement trajectories exclusive of head movement (Fig. 1).

### Data Analysis of Kinematic Measures

From each chewing sequence, four variables were extracted from the jaw movement recordings data using a custom MATLAB program [26]: (1) duration of the chewing sequence, (2) number of cycles in the chewing sequence, (3) 3D working space of movements (mm^3^), and (4) cycle-to-cycle spatiotemporal variability. These variables were chosen because they are expected to change with disease progression [27] and because they parallel commonly used observation-based clinical metrics of chewing performance.

#### Temporal Measures

**Fig. 2:**
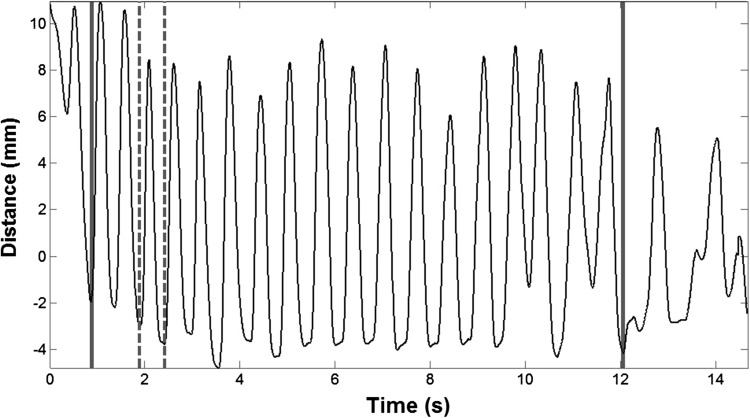
A time history of the distance between the head and the mandible during chewing. Chewing sequences were parsed to exclude extraneous movements such as placement of the food bolus. Only the portion between the solid lines was used for the kinematic analysis. From the time history, individual chewing cycles were identified and used to calculate the STI. The portion between the *dashed lines* indicates the onset and offset of the chewing cycle

The onset and offset of each chewing sequence was defined as the onset of jaw opening for chewing, which was marked by when the spoon was removed from the mouth, to the onset of the first swallow, which was marked by observable laryngeal elevation or lip pursing using both the kinematics and the video as a reference (Fig. 2). These parsing rules were previously used by [Wilson] and colleagues [18, 19]. Although people may have continued to chew after the first swallow, the first swallow was selected as the ending for the chewing sequence to ensure consistency among kinematics and raters as well as to avoid extraneous jaw movements due to clearing of the oral cavity.

**Fig. 3:**
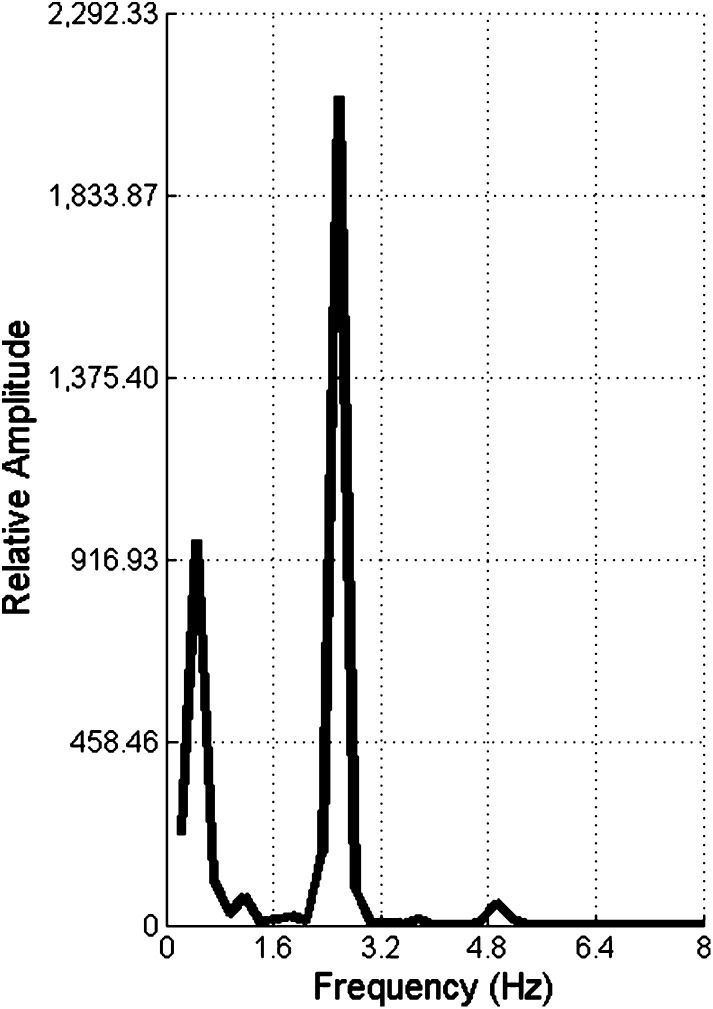
This figure shows the spectral analysis of the chewing sequence. The predominant frequency of each chewing sequence was determined using a fast Fourier transformation. The frequency was then multiplied by the sequence duration to calculate an estimated number of chews

The algorithmic method for computing the number of chewing cycles [19] relied on a fast Fourier transformation (FFT). A Hamming window of 1 s and 1024 points were used for the FFT. The predominant frequency was identified in each sequence (Fig. 3). The predominant frequency represented the rate of chewing, which was then multiplied by the duration of the chewing sequence to provide an estimated number of chewing cycles.

#### Spatial and Spatiotemporal Measures

**Fig. 4:**
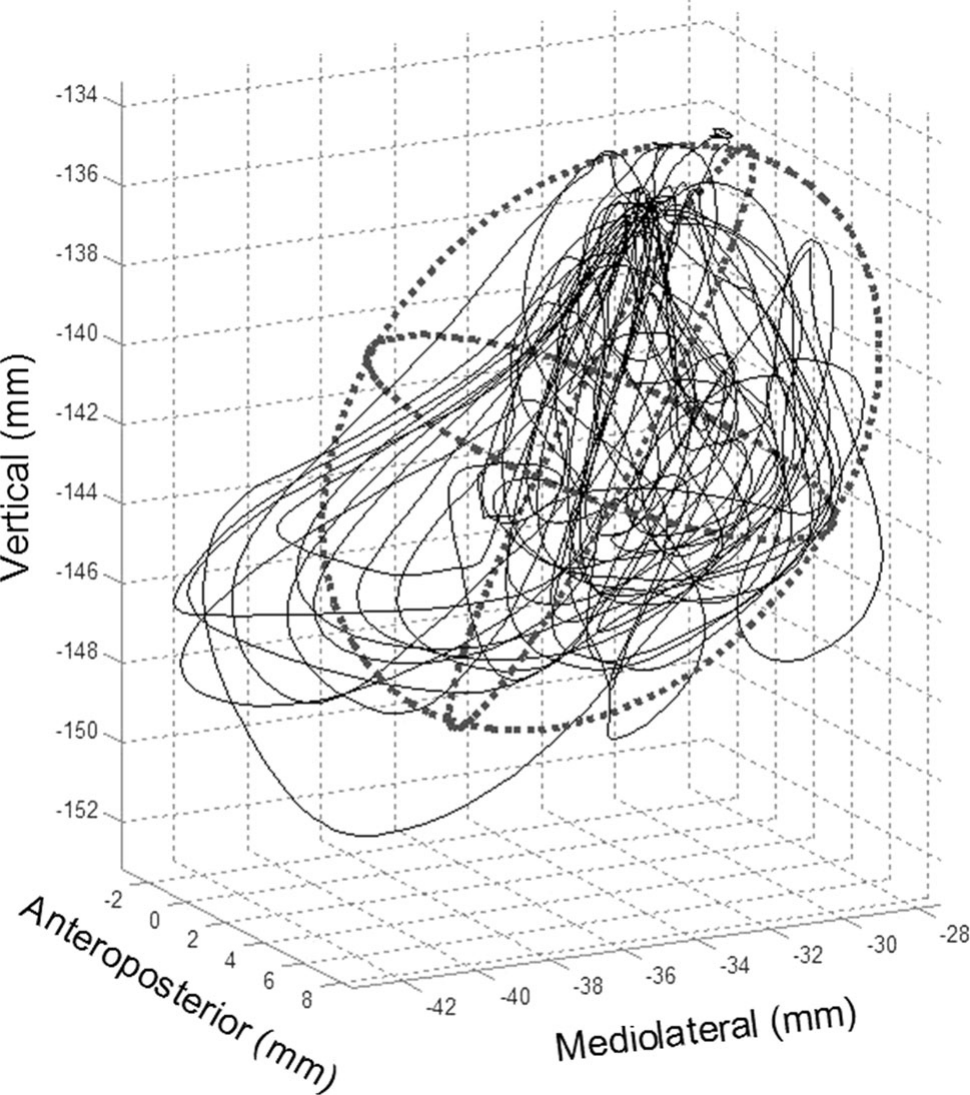
A 3-dimensional representation of the trajectory of jaw motion during chewing. The jaw motion path was fitted with a 2 SD ellipsoid. The volume of the ellipsoid was used to represent the range of motion of the jaw for each chewing sequence

The working space represents the volume (mm^3^) defined by the excursions of the JR marker during the entire chewing sequence. Smaller volumes indicated less overall movement of the jaw. To compute the working space, a two standard deviation (2 SD) ellipsoid was fit around the 3D movement trajectory of the JR marker (Fig. 4). The 2 SD ellipsoid was used to minimize the influence of outliers on the volume calculation.

To quantify jaw movement stability during chewing, the cycle-to-cycle variability of the jaw movement data was calculated by first manually parsing individual chewing cycles.

Because individual chewing cycles can be difficult to identify visually, particularly when chewing is impaired, rules for parsing individual chews were operationally defined. A chewing cycle was defined by an opening and closing phase. A cycle was only included in the analysis, if the opening phase was >25 % of the average vertical amplitude of the chewing sequence. The number of cycles that qualified as chews was counted to calculate the total number of chewing cycles in each sequence. Using this criterion, extraneous movements of the jaw not associated with a chewing cycle were excluded. A trained research assistant parsed all of the files. The intra-rater reliability (for 10 chewing sequences) for the number of chewing cycles in each sequence was *r* = 1.00, 95 % CIs (1.00–1.00)*, p* < 0.001 and the intra-rater reliability for the amplitude and duration of each chewing cycle was *r* = 0.99, 95 % CIs (0.96–0.99)*, p* < 0.001 and *r* = 0.97, 95 % CIs (0.88–0.99)*, p* < 0.001, respectively. The number of chewing cycles included in the analyses varied with each sequence with a mean of 12.22 (6.83) and a range of 3–28 cycles.

The spatial temporal index (STI), a measure of the spatiotemporal movement pattern consistency across repeated trials, was used to determine the consistency of each chewing sequence. The individual cycles for each sequence were time and amplitude normalized and divided into 2 % intervals. The standard deviations were calculated for each interval and then summed to represent the STI [28]. Chewing sequences with lower STIs were judged to be more stable than sequences with higher STIs.

### Statistical Analysis

The intra class correlation coefficient (ICC 2,1) was used to assess the reliability of the ratings of the five SLPs for the four kinematic variables for the videos of the neuro-typical controls and participants with ALS. An ICC of 0.81–1.00 is considered very good, 0.61–0.80 is considered good, 0.41–0.60 is considered moderate, and below 0.40 is considered poor [29]. A Pearson’s correlation was used to assess the validity between the kinematic analysis and the SLPs’ estimates for each measure for both groups of videos. A correlation of 0.70–0.90 is considered strong, 0.40–0.60 is considered moderate, and 0.10–0.30 is considered weak [30]. The mean of the SLP ratings was correlated with the corresponding kinematic measures for each of the videos. The SLP’s responses to the rating questions were treated as continuous variables. The algorithmic method using kinematics for estimating the number of chewing cycles in the sequence was correlated with the manual approach using a Pearson’s correlation. To ensure the chewing sequences with less than 5 cycles did not skew the STIs towards a lower value, the number of cycles and STI value were correlated using a Pearson’s Correlation. R Development Core Team [31] was used for statistical analysis.

## Results

### Duration of the Chewing Sequence

#### Inter-rater Reliability

All 5 SLPs demonstrated very good inter-rater reliability when estimating the duration of the sequence for both the videos of the neuro-typical controls and participants with ALS, ICC = 0.96, *p* < 0.001 and ICC = 0.98, *p* < 0.001, respectively.

#### Validity

**Fig. 5:**
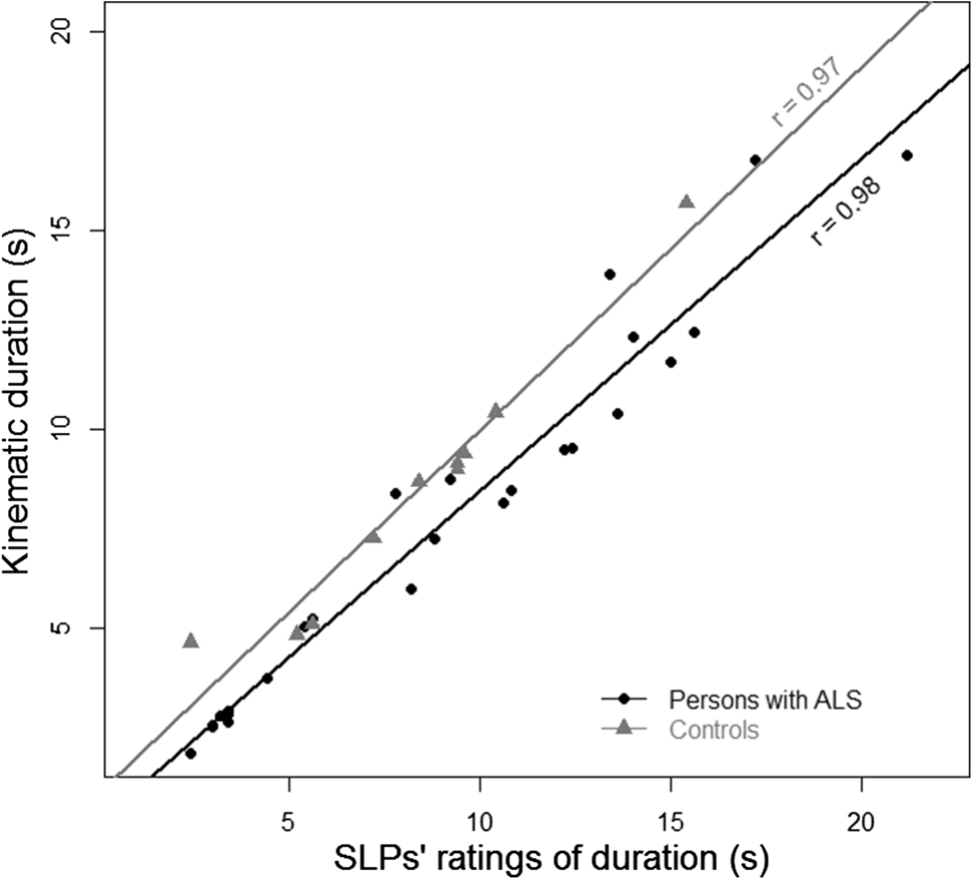
The correlation between the mean of the SLPs’ ratings and the kinematic analysis of the chewing sequence duration for the participants with ALS and the neuro-typical controls

The mean of the SLPs’ estimates correlated with the kinematic analysis of duration is plotted in Fig. 5. The mean of the SLPs’ estimated durations for control and ALS videos was strongly correlated with the kinematics, *r* = 0.97, 95 % CIs (0.89–0.99)*, p* < 0.001 and *r* = 0.98, 95 % CIs (0.94–0.99)*, p* < 0.001, respectively.

### The Number of Chewing Cycles in the Sequence

#### Inter-rater Reliability

The inter-rater reliability among the 5 SLPs for the control videos was moderate for the number of chewing cycles in the sequence, ICC = 0.53, *p* < 0.001 and was good for the ALS videos, ICC = 0.67, *p* < 0.001. As the number of chewing cycles increased, the difference between the SLP’s ratings also increased.

#### Validity

**Fig. 6:**
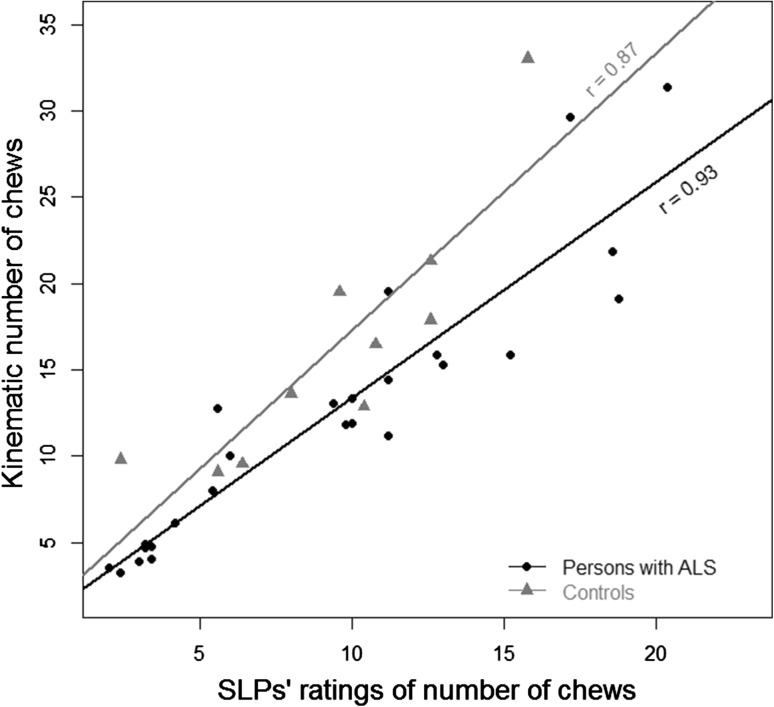
The correlation between the mean of the SLPs’ ratings of the number of chews in each sequence and the kinematic analysis for the participants with ALS and the neuro-typical controls

The mean estimated number of chewing cycles for all 5 SLPs was strongly correlated with the kinematics using the algorithmic approach*, r* = 0.87, 95 % CIs (0.51–0.97), *p* < 0.001 for the control videos and *r* = 0.93, 95 % CIs (0.84–0.97), *p* < 0.001 for the ALS videos. Figure 6 shows a scatter plot of the correlation between the SLPs’ estimates and the kinematic analysis for both sets of videos. The number of chewing cycles for all the videos using the algorithmic approach was strongly correlated with the number of chewing cycles using the manual approach; the latter approach was used when parsing individual cycles to calculate the STI*, r* = 0.93, 95 % CIs (0.86–0.96), *p* < 0.001.

### Range of Motion of the Jaw During Chewing

#### Inter-rater Reliability

The inter-rater reliability among the 5 SLPs for the control videos was poor, ICC = 0.35, *p* = 0.002 and the inter-rater reliability for the ALS videos was moderate, ICC = 0.52, *p* < 0.001.

#### Validity

**Fig. 7:**
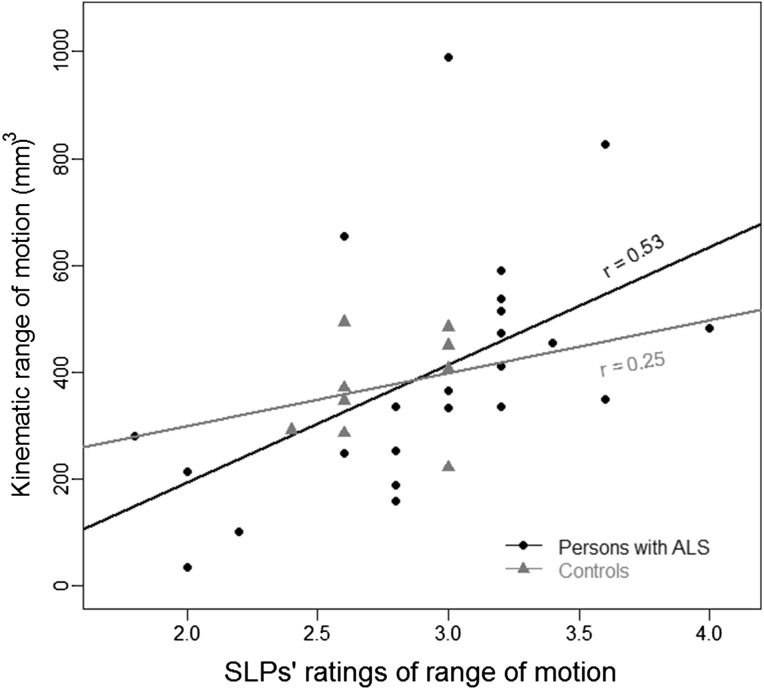
The correlation between the mean of the SLPs’ ratings and the kinematic analysis of the range of motion of the jaw for the participants with ALS and the neuro-typical controls. One outlier was removed from each group and the correlation coefficients reflect the analyses without the outlier

The mean of the estimated range of motion of the mandible for the control videos was strongly correlated with the kinematic measure of working space, *r* = 0.83, 95 % CIs (0.43–0.96), *p* = 0.002, although this correlation was most likely inflated by one outlier that was greater than 2.5 SD from the mean. This correlation became weak when the outlier was removed, *r* = 0.25, 95 % CIs (−0.49 to 0.78), *p* = 0.51 (Fig. 7). For the ALS videos, the SLPs’ estimates were moderately correlated with the kinematic measures, *r* = 0.67, 95 % CIs (0.37–0.84), *p* < 0.001 (Fig. 7). When one outlier, greater than 2.5 SD, was removed, the estimates remained moderately correlated with the kinematic measures, *r* = 0.53, 95 % CIs (0.16–0.78), *p* = 0.009.

### Consistency of the Chewing Pattern

#### Inter-rater Reliability

The inter-rater reliability among the 5 SLPs was good for both the control and ALS videos, ICC = 0.63, *p* < 0.001 and ICC = 0.63, *p* < 0.001, respectively.

#### Validity

**Fig. 8:**
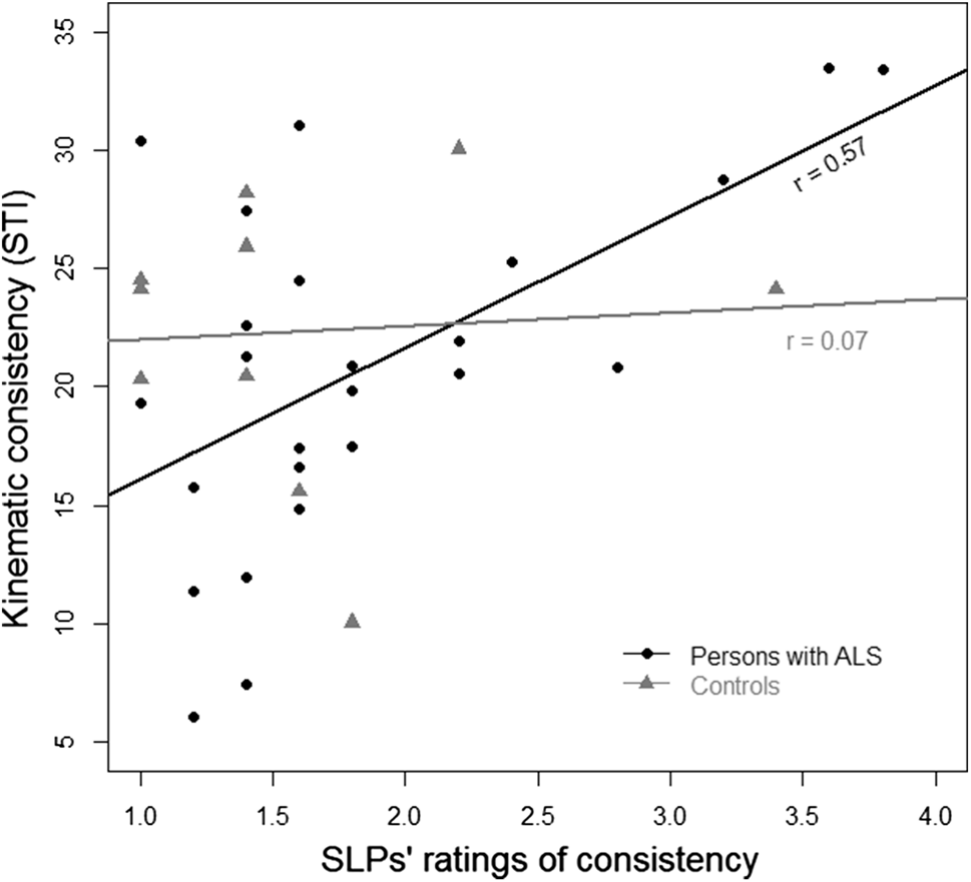
The correlation between the mean of the SLPs’ ratings of the consistency of the chewing pattern and kinematic analysis for the participants with ALS and the neuro-typical controls. For the kinematic analysis, a larger STI reflects an inconsistent chewing pattern and for the SLPs, a rating of “4” reflects a severely inconsistent chewing pattern

The mean estimates of the SLPs for the control videos were poorly correlated with the kinematic measure of the spatiotemporal variably using the STI*, r* = 0.07, 95 % CIs (−0.58 to 0.67), *p* = 0.85 (Fig. 8). The SLPs’ estimates for the ALS videos were moderately correlated with the kinematic measure, *r* = 0.57, 95 % CIs (0.23–0.79), *p* = 0.002 (Fig. 8). To ensure the number of chewing cycles did not affect the STI value, a correlation between the number of cycles in a sequence and STI was calculated. The resulting nonsignificant, weak correlation, *r* = 0.24, 95 % CIs (−0.10 to 0.53), *p* = 0.17, suggests that the variation across participants in the number of cycles included in the STI calculation did not systematically influence the results of the analysis.

## Discussion

The results suggest that the inter-rater reliability and validity of clinical ratings of chewing performance varied across measures. Specifically, ratings of the temporal aspects of chewing (i.e., number of chewing cycles in a sequence and the chewing sequence duration) were reliable and valid; whereas, the efficacy of clinical ratings of spatiotemporal aspects of chewing (i.e., range of motion of the jaw and consistency of the chewing pattern) was weak because of questionable inter-rater reliability and validity.

### Clinician Ratings of Temporal Aspects of Chewing were Reliable and Valid

The high reliability and validity for number of chewing cycles and duration of the chewing sequence supports the efficacy of these measures as diagnostic indicators of chewing impairment in persons with ALS. These findings are consistent with prior studies that investigated the reliability and validity of observation-based estimates of chewing in adults [32, 33]. In young children, Gisel [34] found that raters had high agreement when evaluating the duration and number of chewing cycles; and recommended that clinicians use these parameters when assessing children with feeding disorders. Visual observation may be an adequate level of granularity for evaluating the temporal aspects of chewing because the onset and offset of the chewing sequence can be reliably determined. Similarly, the prominent oscillations of the mandible may provide robust cues for counting chewing cycles.

### The Efficacy of Clinician Ratings for Assessing the Spatial and Spatiotemporal Measures is Questionable

The reliability or validity of clinical ratings for (1) range of motion and (2) movement pattern consistency were questionable. Inter-judge reliability of range of motion was moderate for the videos of persons with ALS and poor for the control videos. Clinicians may have had difficulty discerning differences in the range of motion among the neuro-typical controls because, as a group, they exhibited smaller variations in their range of motion (as evidenced by the kinematic analysis) than did the group with ALS (See Fig. 7).

Despite the good inter-judge agreement for the chewing pattern consistency ratings, the correlation between these ratings and the associated kinematic measure, spatiotemporal variability, was weak. This rating may be particularly vulnerable to observational error because small deviations in the spatial aspects of mandibular movements over the course of a chewing sequence are likely to be difficult to discern visually. By contrast, the kinematic analyses were ideally suited for quantifying even small cycle-to-cycle fluctuations in chewing movement patterns. The raters in this study uniformly indicated that it was very difficult to evaluate the chewing pattern consistency suggesting that judges even with considerable experience may have difficulties detecting normal from abnormal deviations in jaw movement patterns across chewing cycles. Moreover, movement of other facial structures, such as lips, cheeks, and the tongue clearing the oral cavity, may make it difficult for a clinician to focus solely on jaw movements and “may overshadow the visualization of jaw movements” [18, p. 310]. Judgments may also vary depending on which anatomic plane is being visualized during assessment [18]. For example, a sagittal plane view may limit a clinician’s ability to detect variations in the horizontal rotary aspect of chewing.

## Limitations

The inter-judge reliability scores may have been inflated because the SLPs were allowed to view the videos as many times as needed to complete the ratings, which is often not possible in clinical settings where judgments are made on-line. In addition, because SLPs were only provided with a facial plane view on video, features of movement that were predominantly in the sagittal plane may have been undetected. Finally, this study included 5 raters, all of whom met the minimum requirements to investigate the preliminary questions of reliability and validity of these metrics. Because of the small number of raters, the role of experience and training could not be addressed but would be important for future studies.

## Future Directions

Although the high reliability and validity for some temporal aspects of chewing supports their clinical use, additional studies are needed to determine their sensitivity and specificity for identifying ALS-related chewing impairments. An important next step is also to determine what measures of mastication decline with disease progression, and how the changes affect swallowing safety and the nutritional and health status of individuals with ALS. For the temporal measures that were found to be reliable, it is important to continue to develop standardized assessment protocols.

In the future, 3D motion capture technology may be a better option for assessing chewing performance. Motion capture technology has been successfully used in the field of physical therapy and sports performance for many years [35] and systems are rapidly becoming affordable for face tracking (e.g., Microsoft Kinect) making it feasible for wide-scale clinical use [36].

## Conclusions

In this study, temporal measures (i.e., duration of the chewing sequence, number of chewing cycles) were shown to have strong inter-rater reliability and correlated well with the kinematic analysis rendering them appropriate for clinical application. The reliability and validity for spatial and spatiotemporal measures (i.e., range of motion of the jaw, consistency of the chewing pattern) were not as strong, and other assessment methods besides clinical observations should be explored. Reliable descriptions about the changes in jaw performance for chewing may not only provide important information for assessment and disease monitoring, but will also inform our understanding of disease progression.
